# 

**Published:** 1895-03-02

**Authors:** 


**Notices of Births, Deaths, and Marriages** are inserted in this column at a charge of 2s. 6d. for an announcement not exceeding four lines.

**Notice to Advertisers and Subscribers.**

All Advertisements, Orders for Copies of The Hospital and Business Communications should be addressed to **The Manager, NOT TO THE EDITOR,** The Hospital, 428, Strand, London, W.C.

The Cost of Subscription, post free, is as follows:—Per week, 2½d.; per quarter, 2s. 8d.; per half-year, 5s. 3d.; per annum, 10s. 6d. Foreign:—Per Annum, 15s. 2d.; payable, in all cases, in advance.

### NOTICE TO CORRESPONDENTS.

All MS., Letters, Books for Review, and other matters intended for the Editor should be addressed The Editor, The Lodge, Porchester Square, London, W.

The Editor cannot undertake to return rejected MS., even when accompanied by stamped directed envelope.

**"Burdett's Hospital Annual," 1895,**

Ready Early in March.

Sixth year of publication. About 1000 pp. Scarlet cloth, gilt lettered. Price 5s. A most complete guide to British, Colonial, Indian, and American Hospitals, Dispensaries, Nursing and Convalescent Institutions, and Asylums. A few copies of the "Annual" for 1894 may still be obtained on application to the Publishers, The Scientific Press (Limited), 428, Strand, London, W.C.

**NOTICE.—The Index for Vol. XVI. (ending September 24th, 1894) is on sale at the Office of this Journal, Price 2½d., post free.**

### SCRAPS AND GLEANINGS.

IN America a woman, Dr. Anna Williams, has been appointed a Bacteriologist of the New York Board of Health.

DURING the recent epidemic of small-pox in Dublin none of the attendants in the hospitals have been attacked in a fatal manner.

HER Majesty has sent an annual subscription of £10 in aid of the funds of the Royal National Hospital for Consumption at Ventnor.

THE Bulwell (Nottingham) Finishing Company have raised £40 this year in weekly contributions, amongst the working classes, towards the funds of the local hospitals.

HER Majesty has forwarded £250 to the Metropolitan Visiting and Relief Association, through the Bishop of London, to assist in alleviating the present distress in the poorer parts of London.

THE total amount already promised in subscriptions to the Southport New Infirmary building fund is £15,000. It is estimated that about £3,500 will still be required to complete the work.

DR. JOSE COUTINO, professor in the Medico-Chirurgical School of Oporto, died at Lisbon recently. He was physician-in-ordinary to the late King Luis I., and held several other honourable posts.

FIELD hospital work in India is severe. We hear of a hospital and its service, having arrived off a journey, being required to move again in the middle of the night, covering forty-five miles in the second transit.

A SPECIAL appeal is going to be made by the authorities of the Sidmouth Cottage Hospital to the local ministers of all denominations with a view to the income being increased and the adverse balance cleared off.

A SATISFACTORY report has been presented by the committee of the Newcastle Infirmary. The question of a new site has again forcibly presented itself, the building now existing being very old and unfit in many ways for the present requirements.

PROFESSOR RITTER VON KRAUS has received the prize offered by the Austrian Temperance Society for the best essay showing how the cure of the growing abuse of alcoholic stimulants can be counteracted in schools. The prize thus gained by the Professor was 500 crowns.

M. ROUX'S method of anti-toxin inoculation for diphtheria has been ordered official study at Uruguay, the Republic having authorised Dr. J. Roderquez and Dr. Turenne to study the same in furtherance of M. Roux's work. At Versailles the same orders will also be carried out.

THE Ladies' Auxiliary Committee, in connection with the Cancer Hospital at Glasgow, have collected no less a sum than £227 in aid of the charity. A site for the new hospital has at length presented itself, and it is hoped the authorities will soon be able to commence the work of extension.

A VERY important proposal was laid before the governors of the Bedford General Infirmary; this amounted to no less a project than the building of a new hospital for Bedford, the existing infirmary failing to reach a reasonable standard, being defective in arrangement from basement to roof.

AT the annual meeting of the London Fever Hospital, on Friday, the report showed that scarlet fever had been much less prevalent last year than during 1893. The question of rebuilding the hospital has been under consideration, and the construction of the new laundry will be undertaken at once.

MR. WHITBREAD, M.P., and the Duke of Bedford have each promised £5,000 towards the building fund of the Bedford General Infirmary. A further sum of £20,000 is required to carry out the scheme, and it is suggested that the same be raised, if possible, without touching the invested funds of the institution.

WE hear rumours of a renewed activity with regard to an isolation hospital for Richmond. The existing friendly arrangements between the Richmond Corporation and the Heston and Isleworth District Council is of great value to both parties. It is only too clearly to the ratepayers' advantage to carry out a combined action.

A MEETING has taken place at the Hartley Institution, Southampton, to consider the question of raising the necessary funds for carrying out a scheme and plans for the proposed alterations and additions to the Royal South Hants Infirmary, as presented to the governors of the institution by Messrs. Young and Hall, the sanitary architects.

THE necessity for increased hospital accommodation in Manchester is a steadily-growing problem, and the question in the city as to how this may best be solved presents itself from time to time. Four large hospitals already are under the Infirmary Board, and it is satisfactory to learn that the labours of last year were carried out most successfully in all departments.

A VERY heavily-debited balance-sheet is presented in the annual report of the Royal Southern Hospital at Liverpool. At the end of last year this had reached to over £5,000, and the committee appealed for help to remove the load which hampered their work, and which, if not removed, would compel them to close some of the wards. The accommodation of the wards had been taxed to the uttermost during the year.

### THE STUDENT OF MEDICINE.

### APPOINTMENTS.

G. F. Bodington, F.R.C.S., M.R.C.P., appointed Medical Superintendent to the New Westminster Asylum, Canada. W. G. Davies, L.R.C.P., L.R.C.S Edin., appointed Medical Officer for the Western District of the Llandilo Rural District Council. Mr. A. De la Touche, appointed Medical Officer for the Soothill Nether District of the Dewsbury Union. Mr. L. H. D. Hale, appointed House Surgeon to the Boscombe Hospital and Provident Dispensary. G. Hancock, M.R.C.S.Eng., L.R.C.P.Lond., appointed Obstetric House Physician to Westminster Hospital. W. J. Harper, M.R.C.S., L.R.C.P., appointed Medical Officer and Public Vaccinator for the Braunton District of the Barnstaple Union. J. Johnston, M.D.Edin., appointed Public Vaccinator for the Western District of the Bolton Union. J. H. Keay, M.A.Edin., appointed Medical Officer for the Colne District of the Burnley Union. Mrs. Caroline Keith, L.R.C.S., L.R.C.P.Edin., appointed Anæsthetist to the Chelsea Hospital for Women. E. Knight, M.R.C.S., L.R.C.P., appointed House Surgeon to the Salford Royal Hospital. Mr. C. G. Max-Vickers, appointed Medical Officer for the No. 8 District of the Newmarket Union. J. H. Ray, M.B., Ch.B.Vict., M.R.C.S., L.R.C.P., appointed Junior House Surgeon to the Salford Royal Hospital. J. R. I. Raywood, L.R.C.P.Lond., M.R.C.S.Eng., appointed Honorary Surgeon to the Montgomeryshire Infirmary. Mr. E. A. M. Roberts, appointed Surgeon to the Penrhyn Quarry Hospital. F. G. Robinson, M.B.Vict., L.R.C.P.Lond., reappointed District Medical Officer to the Salford Royal Hospital. S. J. Stuck, M.R.C.S., L.R.C.P., appointed Resident Medical Officer

**THE STUDENT OF MEDICINE**—*continued.*

to the Chelsea Hospital for Women. T. P. G. Wells, L.R.C.P. Edin., L.R.C.S.Edin., L.F.P.S.Glasg., appointed Honorary Medical Officer to the St. Albans Hospital and Dispensary. Mr. Williams, appointed Medical Officer for the Crowland District of the Peterborough Union. J. F. Wright, M.R.C.S.Eng., appointed Medical Officer for the Western District of the Bolton Union.

**VACANCIES.**

The following vacancies are announced this week, the amount of salary in each case being given after the name of the institution:

**Resident Medical Officers.**

North London Consumption Hospital, Hampstead, N.W.—Medical Officer; doubly qualified. Appointment for one year, but eligible for re-election. Honorarium £40 per annum, with board, rooms, &c. Applications to the Secretary, Lionel F. Hill, M.A., by March 4th.

Northumberland County Lunatic Asylum, Morpeth.—Assistant Medical Officer; unmarried. Salary £120 per annum, increasing £10 yearly to £150, with furnished apartments, board, and lodging. Applications (marked outside "Medical") to Dr. McDowell at the Asylum by March 11th.

Queen Charlotte's Lying-In Hospital, Marylebone Road, N.W.—Resident Medical Officer. Appointment for four months. Salary at the rate of £60 per annum, with board and residence. Applications to the Secretary by March 2nd.

Royal Surrey County Hospital, Guildford.—Assistant House Surgeon. Board, residence, and laundry found. No salary. Applications to the Honorary Secretary by March 4th.

St. George's and St. James's Dispensary, 60, King Street, Golden Square, W.—Resident Medical Officer. Salary £100 per annum, with furnished apartments, fuel, light, and attendance. Applications to S. Leger Burnett, Secretary, by March 2nd.

**Non-Resident Medical Officers.**

Cape Colony.—Medical Officer of Health for Cape Colony. Salary £1,000 per annum. Applications to the Agent General, 112, Victoria Street, S.W., or the Colonial Under-Secretary, Cape Town, by March 15th.

**UNIVERSITIES AND COLLEGES.**

**University of Cambridge.**

Anthropology.—Professor Macalister announces a new course of lectures with practical work in anthropology, to be given by Mr. A. C. Haddon on Thursday during the Lent and Easter terms.

Dr. Dohrn's Station.—Mr. M. Laurie has been appointed to occupy the University's table in the Naples Zoological Station for three months from March 1st.

Honorary Degree.—The degree of Sc.D. *honoris causâ* is to be conferred on Sir William McGregor, M.D. Aberd., K.C.M.G., Administrator British New Guinea, in recognition of his able services to ethnography.

Electors.—Dr. Allbutt has been appointed an Elector to the Chair of Anatomy, Dr. A. Macalister to the Chairs of Medicine (Downing) and Surgery, Dr. Gaskell to the Chair of Pathology, Dr. D. MacAlister to the Chair of Zoology.

**Royal College of Physicians of Ireland.**

On Friday, February 1st, the President admitted to the Licences in Medicine and Midwifery the following candidates who have been successful at the Final Examination held in January, 1895, under the Conjoint Scheme with the Royal College of Surgeons in Ireland: A. St. L. Burke, T. Cairns, M. A. Corcoran, M. Delany, J. Fleming, W. A. Gordon, R. D. Jephson, T. J. Jordan, H. G. Martin, W. F. A. M'Cann, W. Stratton, J. H. Walsh. Mr. G. S. Davidge, who had passed the Final Professional Examination in October, 1894, but who was not then of age, was also admitted. Mr. J. H. J. L. Davys, not yet being of age, was not admitted, although he had passed the Final Professional Examination in January.

